# Escin Sodium Improves the Prognosis of Acute Pancreatitis via Promoting Cell Apoptosis by Suppression of the ERK/STAT3 Signaling Pathway

**DOI:** 10.1155/2021/9921839

**Published:** 2021-08-12

**Authors:** Qian Zhang, Chen Zhao, Lei Zhang, Kai Sun, Linlin Yu, Xianming Wang, Lei Ren, Nan Zhang, Chengyu Chen, Ju Liu, Haimei Wang, Hu Tian

**Affiliations:** ^1^Department of General Surgery, Shandong Provincial Qianfoshan Hospital, Shandong University of Traditional Chinese Medicine, China; ^2^Weihai Hospital Affiliated to Shandong University of Traditional Chinese Medicine, No. 29, Qingdao North Road, Huancui District, Weihai City Shandong Province, China; ^3^College of First Clinical Medicine, Shandong University of Traditional Chinese Medicine, 16369 Jingshi Road, Lixia District, Jinan City, Shandong Province, China; ^4^Department of General Surgery, The First Affiliated Hospital of Shandong First Medical University & Shandong Provincial Qianfoshan Hospital, No. 16766, Jingshi Road, Jinan City, Shandong Province, China; ^5^Shandong Hospital of Traditional Chinese Medicine Affiliated to Shandong University of Traditional Chinese Medicine, 16369 Jingshi Road, Lixia District, Jinan City, Shandong Province, China; ^6^Department of Thoracic Surgery, The First Affiliated Hospital of Shandong First Medical University & Shandong Provincial Qianfoshan Hospital, No. 16766, Jingshi Road, Jinan City, Shandong Province, China; ^7^Laboratory of Microvascular Medicine and Medical Research Center, Shandong Provincial Qianfoshan Hospital, The First Affiliated Hospital of Shandong First Medical University, No. 16766, Jingshi Road, Jinan City, Shandong Province, China

## Abstract

Acute pancreatitis (AP), an inflammatory disorder of the pancreas, can cause systemic inflammatory responses. Escin Sodium (ES), a natural mixture of triterpene saponins extracted from the dry ripe fruit of Fructus Aesculi or horse chestnut crude, has been demonstrated to have antiedematous, anti-inflammatory, and antiexudative effects. We here aim to investigate the effects of ES pretreatment on AP in vivo and in vitro and explore its potential molecular mechanism. In the present study, we demonstrated that ES pretreatment could apparently decrease amylase and lipase, downregulate inflammatory cytokines, and attenuate pancreatic damage. Additionally, the increased expression of apoptotic-related proteins and the results of flow cytometry demonstrated the effects of ES on promoting apoptosis in acinar cells. Moreover, ES could enhance mitochondrial membrane potential (MMP, *ΔΨ*m) and reactive oxygen species (ROS) level and reduce intracellular calcium concentration, which are closely related to mitochondrial-mediated death. The effect of ES pretreatment on acinar cell apoptosis was furtherly confirmed by the regulatory pathway of the ERK/STAT3 axis. These results suggest that ES attenuates the severity of AP by enhancing cell apoptosis via suppressing the ERK/STAT3 signaling pathway. These findings provide evidence for ES which is treated as a novel and potent therapeutic for the treatment of AP.

## 1. Introduction

Acute pancreatitis (AP)—an inflammatory disorder of the pancreas—is the leading cause of admission to hospital for gastrointestinal disorders in many countries [1] and has become an economic burden to patients and health care system [2]. According to the revision of the Atlanta (2012) [3], the classification of AP defines three degrees of severity: mild acute pancreatitis, moderately severe acute pancreatitis, and severe acute pancreatitis (SAP). SAP is characterized by persistent organ failure that results in multiple organ dysfunction syndrome (MODS) and systemic inflammatory response syndrome (SIRS) by activation of cytokine cascades during the early phase [4–7]. Patients who develop MODS or SIRS within the first few days are suffering from the increased risk of death, with mortality reported to be as great as 36–50% [4, 5, 7]. At present, many theories such as pancreatic self-digestion, inflammatory responses, oxidative stress, and calcium overloaded and cell apoptosis are supposed to explain the underlying mechanism of AP. However, the pathogenesis of AP is complicated and has not been elucidated completely yet.

The outcome of AP is determined by the type of acinar cell death. The type of cell death is as follows: apoptosis, necrosis, autophagy, necroptosis, pyroptosis, etc. [8]. As studies showing, the severity of acute pancreatitis is correlated with necrosis and inversely with apoptosis [9]. A shift of necrosis to apoptosis can ameliorate the prognosis of acute pancreatitis. The management of AP covers supportive care (e.g., isotonic intravenous fluids and pain control) and nutrition [10]. Although researchers have made great efforts in acute pancreatitis, there is still no specific drug for it.

**Figure 1:**
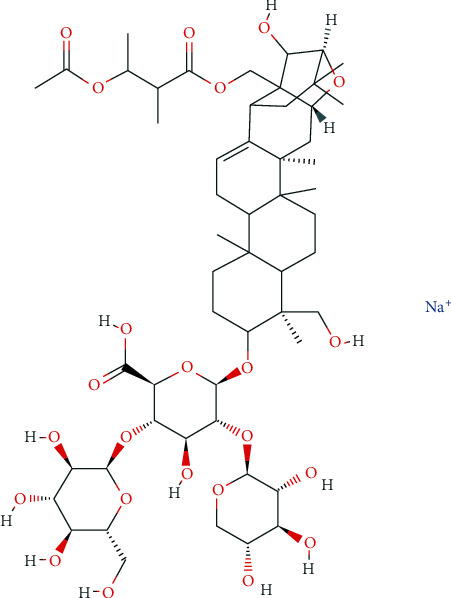
Chemical structure of Escin Sodium.

Active compounds of many plants are used for medical demands and provide additional therapeutic effects for modern medicine [11]. Many agents currently used in the clinic are from natural plants widely, such as artemisinin, aspirin, taxanes, and Vinca alkaloids. Escin Sodium (ES), C55H83NaO23 (Figure 1), a natural mixture of triterpene saponins extracted from the dry ripe fruit of Fructus Aesculi or horse chestnut crude [12–15], was used as medicine for centuries. ES has been demonstrated to have antiedematous, anti-inflammatory, and antiexudative effects [12, 15], having shown excellent tolerability [15]. Recent studies have identified that ES can induce apoptosis in various diseases, such as human osteosarcoma [16], renal cancer cells [17], human acute leukemia Jurkat T cells [15], and hepatocellular carcinoma [13]. However, the role of ES on AP and relevant mechanism still remains unclear.

ERK—an important molecule in the classical MAPK cascade pathway, regulating many cell functions, including apoptosis, proliferation, and migration [18, 19]—can widely activate downstream molecules. STAT3 (signal transducer and activator of transcription), one of the downstream of ERK, is also linked with survival [20]. Studies have shown that phosphorylation of ERK can drive phosphorylation of STAT3 [21]. But it is still unknown whether ERK/STAT3 gets involved in the process of AP.

In this study, we demonstrated that ES can optimize the prognosis of acute pancreatitis. We furtherly explored the underlying mechanisms and acquired the results indicating that ES promotes apoptosis in STC-induced acute pancreatitis via downregulating the ERK/STAT3 signaling pathway.

## 2. Materials and Methods

### 2.1. Reagents

Escin Sodium was purchased from Shandong Luye Pharmaceutical Co., Ltd. (Yantai, China), registration number: H200003239. Sodium taurocholate was purchased from Solarbio Science & Technology Co., Ltd. (Beijing, China). PD98059 (ERK1/2 inhibitor) were purchased from MCE (USA). Pentobarbital sodium was purchased from Nanjing Sunshine Biotechnology Co., Ltd. (Nanjing, China).

### 2.2. Animal Experiments

Adult male Sprague Dawley (SD) rats (250-300 g) were purchased from Jinan Pengyue Laboratory Animal Co., Ltd. (Jinan, China). Animal experiments were approved by the Animal Care and Use Committee of the First Affiliated Hospital of Shandong First Medical University (Jinan, China) according to the National Institutes of Health Guidelines for the Care and Use of Laboratory Animals. Rats were housed at room temperature (25°C approximately) with a 12 h light/12 h dark cycle and humidity of 50%. Food and water were provided ad libitum.

A total of 50 male SD rats were randomly divided into 5 groups: control (Con) group treated with 0.9% saline solution, AP group (model group), and 3 groups pretreated with different concentrations of Escin Sodium (1 mg/kg, 3 mg/kg, and 6 mg/kg). Escin Sodium was dissolved in 0.9% saline solution and was intraperitoneally injected 1 h before modeling. Referring to the modeling method of Li et al. [22], rats were anesthetized with 3% pentobarbital sodium and the surgical field was completely exposed. The proximal bile duct was occluded at the hepatic portal by a vascular clamp temporarily, followed by retrograde injection of 5% sodium taurocholate (1 mg/kg body weight) in the distal end of the biliary-pancreatic duct. Changes in pancreatic tissues including edema, hemorrhage, and congestion were observed for 5 minutes. Subsequently, the vascular clamp was removed and sutured the incision. Rats in the control group were anesthetized in the same manner, and 0.9% saline solution was injected into the distal end of the biliary-pancreatic duct.

Animals were sacrificed at 24 h after injection of 5% sodium taurocholate. Blood samples were collected to determine serum digestive enzymes. The pancreatic tissues were removed immediately and washed with cold 0.9% saline solution injection 3 times. Then, the tissues were divided into 3 sections randomly for morphological examination, Western blotting, and real-time reverse transcription (RT) PCR.

### 2.3. Cell Culture and Interventions

AR42J, rat exocrine pancreas cells, were purchased from the Institute of Shanghai Fuheng Cell Biology (Shanghai, China) and were cultured in High-Glucose DMEM (Meilunbio, Dalian, China) containing 20% fetal bovine serum (FBS) (Gibco, United States) and 1% 100 U/mL penicillin and 100 *μ*g/mL streptomycin P/S (Invitrogen, Carlsbad, CA, USA), in a humidified incubator at 37°C with an atmosphere of 5% CO_2_. The AR42J cells (1 × 10^6^ cells/well approximately) were seeded in 6-well plates and washed with D-PBS (PBS without calcium and magnesium) every 48 h before the medium changed. Two hours before intervention, the cells were incubated in a High-Glucose DMEM without FBS and P/S.

AR42J cells were randomly divided into different groups, respectively, as follows:

Control group: cultured with a 2 mL High-Glucose DMEM.

AP group: cultured with a 2 mL High-Glucose DMEM with the concentration of 390 *μ*M STC for 4 h and 8 h, respectively.

AP+Escin Sodium group: cultured with a 2 mL High-Glucose DMEM with the concentration of 10 *μ*g/mL, 20 *μ*g/mL, and 30 *μ*g/mL Escin Sodium for 1 h and then followed by 390 *μ*M STC for 4 h and 8 h, respectively.

AP+PD98059 group: cultured with a 2 mL High-Glucose DMEM with the concentration of 10 *μ*M PD98059 (an inhibitor of ERK1/2 signal) for 1 h and then followed by 390 *μ*M STC for 4 h and 8 h, respectively.

AP+Escin Sodium+PD98059 group: cultured with a 2 mL High-Glucose DMEM with the concentration of 10 *μ*M PD98059 and 20 *μ*g/mL Escin Sodium for 1 h, then followed by 390 *μ*M STC for 4 h and 8 h, respectively.

### 2.4. Histological Analysis and Immunohistochemistry

One portion of pancreatic tissue was selected randomly and fixed in 10% neutral-buffered formalin for 48 h, then embedded in paraffin. The tissues were cut into 4 *μ*m thick sections and stained with hematoxylin and eosin. The assessment of edema, inflammation, vacuolization, and necrosis was performed according to Schmidt et al. [23–25] (Table 1). For immunohistochemistry, paraffin-embedded pancreas sections (4 *μ*m) were heated at 70°C for 2 h and rehydrated by xylene and different concentrations of alcohol. Antigen was retrieved by pressure cooker in 0.01 mol/L citrate buffer at constant 100°C for 2 minutes, then cooled in cold water for 2 hours. Endogenous peroxidase activity was blocked by 3% H_2_O_2_ for 20 min. Having been washed by PBS 5 min/3 times, sections were blocked by 5% normal goat serum for 45 min at room temperature and incubated with rabbit antibodies against MPO (1: 400, Proteintech Group Inc., Wuhan, China), NLRP3 (1 : 50, Proteintech Group Inc., Wuhan, China), GSDMD (1 : 100, Proteintech Group Inc., Wuhan, China), and caspase-1 (1 : 100, Proteintech Group Inc., Wuhan, China) in 4°C overnight. The sections were washed in PBS 5 min/3 times and incubated with a secondary antibody for 1 hour. Then, DAB working solution (Boster Biological Technology Co., Ltd, Wuhan, China) was dropped on the slides and incubated at room temperature for 1 min. Redyeing with hematoxylin was performed for 10 seconds and washing with tap water for 1 min. Finally, all sections were dehydrated and sealed with neutral resin. The slides were observed under a light microscope (×200). Data were analyzed using Image-Pro Plus software 6.0 (Media Cybernetics, Silver Spring, MD, USA).

### 2.5. Biochemical Analysis

Blood samples and cell supernatant were obtained to determine amylase and lipase levels after induction of acute pancreatitis with STC. The serum was centrifuged at 3000g for 10 min at 4°C. The levels of amylase and lipase were analyzed by a colorimetric method using a commercial kit for amylase or lipase (Jiancheng Co., Nanjing, China).

### 2.6. Reverse Transcription- (RT-) PCR and Real-Time- (q-) PCR

Real-time RT-PCR (qRT-PCR) was used to analyze mRNA transcripts in rat pancreas and AR42J cells. The Trizol reagent (Invitrogen, California, USA) was used to isolated total RNA from the pancreas and AR42J cells following the manufacturer's protocol, then converted to cDNA according to the manufacturer's instructions (Vazyme Biotech Co., Ltd, Nanjing, China). Quantitative real-time polymerase chain reaction (PCR) was performed by using SYBR Green SuperMix (Vazyme Biotech Co., Ltd, Nanjing, China) in the CFX96™ Real-time System (Bio-Rad, United States). The primer sequences were as follows:

TNF-*α*: Forward: 5′-GGCGTGTTCATCCGTTCTC-3′

Reverse: 5′-CTTCAGCGTCTCGTGTGTTTCT-3′

IL-6: Forward: 5′-ATTGTATGAACAGCGATGATGCAC-3′

Reverse: 5′-CCAGGTAGAAACGGAACTCCAGA-3′

IL-10: Forward: 5′-CAGACCCACATGCTCCGAGA-3′

Reverse: 5′-CAAGGCTTGGCAACCCAAGTA-3′

IL-1*β*: Forward: 5′-CCCTGAACTCAACTGTGAAATAGCA-3′

Reverse: 5′-CCCAAGTCAAGGGCTTGGAA-3′

IL-18: Forward: 5′-GACTGGCTGTGACCCTATCTGTGA-3′

Reverse: 5′-TTGTGTCCTGGCACACGTTTC-3′

GAPDH: Forward: 5′-GGCACAGTCAAGGCTGAGAATG-3′

Reverse: 5′-ATGGTGGTGAAGACGCCAGTA-3′

Relative mRNA levels were normalized to GAPDH mRNA, and all reactions were performed in triplicate. Melting curve analysis was performed to ensure the specificity of quantitative PCR. Data was performed using the 2^-*ΔΔ*CT^ method for analysis.

### 2.7. Western Blotting

Total protein was extracted from the pancreas and AR42J cells using RIPA (Beyotime, Shanghai, China): cocktail (MCE, USA): phosphatase inhibitors(ApexBIO, Houston, USA) = 100 : 1 : 1(*v*/*v*/*v*). Extraction of cytoplasmic and nuclear protein using the Cytoplasmic & Nuclear protein extraction Kit (Beyotime, Shanghai, China). BCA assay kits (Beyotime, Shanghai, China) were used to detect the concentration of extracted proteins. An equal amount of protein (30 *μ*g) was loaded in each lane and separated by 12.5% (or 10%) sodium dodecyl sulfate-polyacrylamide gel (Epizyme, Shanghai, China). Aim proteins were transferred to PVDF membranes (Millipore, USA) and blocked in 5% nonfat milk for 1 h at room temperature. Having been washed by TBS-T for 5 min/3 times, membranes were probed with the appropriate primary antibodies overnight at 4°C. The primary antibodies were purchased from CST (Cell Signaling Technology, Danvers, MA, USA), including antibodies against phosphor-STAT3 (Tyr705,1 : 2000), STAT3 (1 : 2000), and histone H3 (1 : 1000). Antibodies against Bax (1 : 5000), cleaved caspase-3 (1 : 1000), Bcl-2 (1 : 1000), caspase-1 (1 : 1000), P53 (1 : 1000), and GAPDH (1 : 8000) were from Proteintech (Proteintech Group Inc., Wuhan, China). Antibodies against NLRP3 (1 : 1000), GSDMD (1 : 1000), Anti-ERK1 (phosphor T202)+ERK2 (phosphorT185, 1 : 1000), and ERK (1 : 10000) were from Abcam (UK). Enhanced chemiluminescence reagents (Millipore, USA) were used to detect the bands. The strength was quantified by Image J software, and data analysis was used by GraphPad Prism software 8.0.

### 2.8. Apoptosis Quantified by Flow Cytometry

Cell apoptosis was quantified by the Annexin-V-FITC/PI Apoptosis Detection Kit (Meilunbio, Dalian, China). After the intervention, AR42J cells were digested in Accutase (Mutisciences, Hangzhou, China) and collected subsequently. Having been washed by D-PBS and resuspend in the Annexin V Binding Buffer, 100 *μ*L of cell suspension was transferred into the test tube for staining. 5 *μ*L of Annexin-V-FITC and 5 *μ*L of PI solution were added to each sample, then incubated for 15 min at room temperature in the dark. Having been added 400 *μ*L Annexin V binding buffer to the test tube, cell suspension was texted by the flow cytometry (BD FACSAria™ II Sorter, USA). The results were quantified by FlowJo-V10.0 software.

### 2.9. Hoechst33342 and MitoTracker Red CMXRos Staining

Hoechst33342 and MitoTracker Red CMXRos staining were purchased from Beyotime (Shanghai, China). After intervention, AR42J cells were incubated in a 2 mL High-Glucose DMEM with 20 *μ*L Hoechst33342(10 *μ*mol/L) and 2 *μ*L MitoTracker Red CMXRos staining (200 nmol/L) in each plate for 15 min at 37°C as ordered by the manufacturer's instructions. 2 mL of complete medium was supplemented after being washed twice with D-PBS. The cells were visualized under a Leica confocal laser scanning microscope (EL6000, Wetzlar, Germany). Hoechst33342 was monitored at an excitation wavelength of 346 nm, and MitoTracker Red was monitored at an excitation wavelength of 579 nm to locate nuclear and mitochondria, respectively.

### 2.10. Measure of Intracellular ROS Levels and Calcium Concentration

Levels of intracellular ROS in AR42J cells were detected using ROS assay kits, a chemical fluorescence method (2,7-dichlorodi-hydrofluorescein diacetate, DCFH-DA, Beyotime, Shanghai, China). After the intervention, AR42J cells were incubated in a 2 mL High-Glucose DMEM with 2 *μ*L DCFH-DA (10 *μ*mol/L) for 30 min at 37°C and washed twice in a High-Glucose DMEM, and then 2 mL of complete medium was supplemented. Cells were visualized under a Leica confocal laser scanning microscope (EL6000, Wetzlar, Germany). ROS was monitored at an excitation wavelength of 488 nm to locate reactive oxygen.

Fluo-4-AM was used to detect intracellular calcium concentration (Beyotime, Shanghai, China). After the intervention, AR42J cells were washed 3 times with D-PBS. Then, cells were incubated in 2 mL D-PBS with 1 *μ*L Fluo-4-AM (0.5 *μ*mol/L) for 20 min at 37°C. After being washed by D-PBS, cells were visualized under a Leica confocal laser scanning microscope (EL6000, Wetzlar, Germany). Calcium concentration was monitored at an excitation wavelength of 488 nm to locate.

## 3. Statistical Analysis

The experiments were performed in triplicates and repeated at least three times. Data are analyzed using GraphPad Prism statistical software (version 8, GraphPad Software, Inc., San Diego, CA). Data are represented as means ± standard deviation (SD). Unpaired Student's *t*-test was used to evaluate the significance between 2 groups. For comparison of more than three groups, one-way analysis of variance (ANOVA) was applied. *P* < 0.05 was considered statistically significant.

## 4. Results

### 4.1. Escin Sodium Inhibits Amylase, Lipase, and Inflammatory Cytokines in STC-Induced Acute Pancreatitis

**Figure 2:**
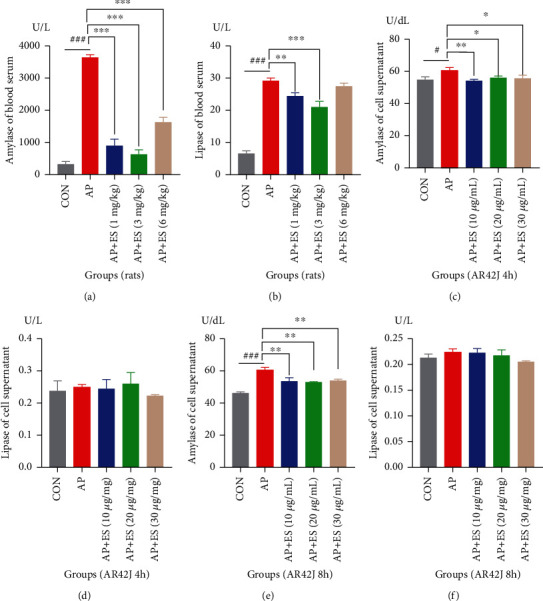
Effects of ES pretreatment on amylase and lipase in vivo and in vitro. Data are expressed as means ± SD. (a) The levels of serum amylase and lipase in the AP group increased compared with the CON (control) group, and ES pretreated groups (1, 3, and 6 mg/kg) decreased amylase significantly. (b) The ES pretreated group (1 and 3 mg/kg) decreased serum lipase. (c, e) Amylase in supernatant increased in STC-induced AP in AR42J cell, and ES pretreated groups (10, 20, and 30 *μ*g/mL) decreased it obviously. (d, f) Lipase was not stimulated by STC, and ES pretreatment has no effect on it (^#^*P* < 0.05, ^##^*P* < 0.01, and ^###^*P* < 0.001 versus the CON group; ^∗^*P* < 0.05, ^∗∗^*P* < 0.01, and ^∗∗∗^*P* < 0.001 versus the AP group).

Elevation of serum amylase and/or lipase levels to at least 3 times the upper limit of normal is a key component of diagnosing acute pancreatitis [26]. To access the effects of ES pretreatment on AP, we measured amylase, lipase, and inflammatory cytokines both in vivo and in vitro. As shown in Figures 2(a) and 2(b), ES remarkably downregulated serum amylase, lipase in vivo. Amylase of cell supernatant decreased significantly in ES pretreatment groups (Figures 2(c) and 2(e)); in contrast, there was no significant change in lipase (Figures 2(d) and 2(f)).

**Figure 3:**
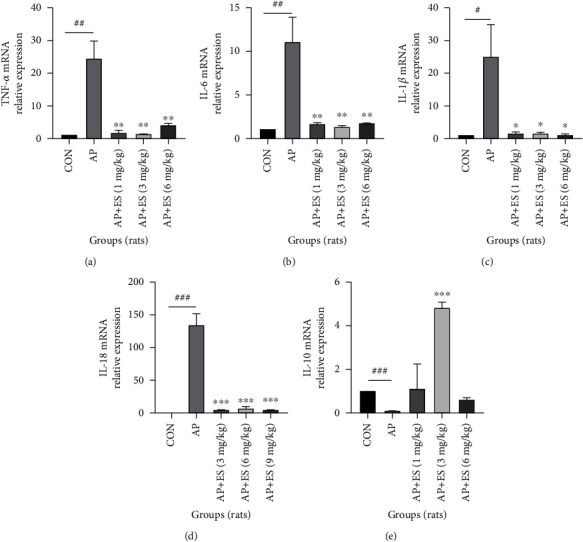
Effects of ES on inflammatory cytokines in vivo. The data were presented as the mean ± SD of three replicate experiments. (a–d) Serum levels of TNF-*α*, IL-6, IL-1*β*, and IL-18 in the AP group were higher than those in the control group, and ES pretreated with different concentrations decreased them significantly. (e) IL-10 decreased obviously in the AP group but increased in the ES (3 mg/kg) pretreatment group (^#^*P* < 0.05, ^##^*P* < 0.01, and ^###^*P* < 0.001 versus the CON group, ^∗^*P* < 0.05, ^∗∗^*P* < 0.01, and ^∗∗∗^*P* < 0.001 versus the AP group).

**Figure 4:**
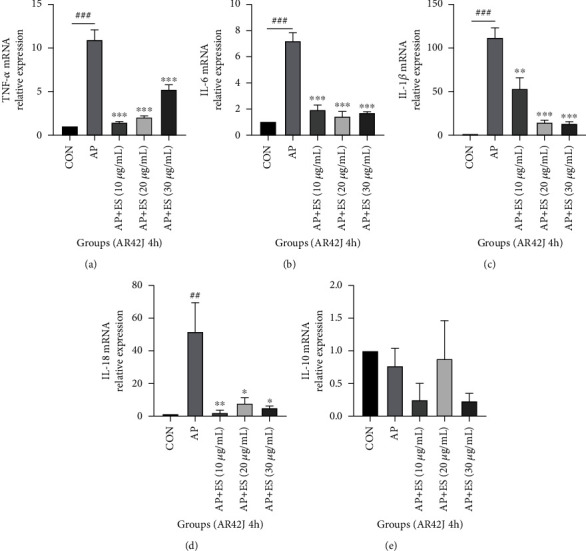
ES pretreatment inhibits inflammatory cytokines in vitro. AR42J cells were pretreated with different concentrations of ES for 1 h, then stimulated with STC for 4 h or 8 h. The data were presented as the mean ± SD of three replicate experiments. (a–d) Levels of TNF-*α*, IL-6, IL-1*β*, and IL-18 increased significantly in the AP group, and ES with different concentrations could downregulate them. (e) There was no significant change in IL-10 in each group stimulated for 4 h (^#^*P* < 0.05, ^##^*P* < 0.01, and ^###^*P* < 0.001 versus the CON group, ^∗^*P* < 0.05, ^∗∗^*P* < 0.01, and ^∗∗∗^*P* < 0.001 versus the AP group).

**Figure 5:**
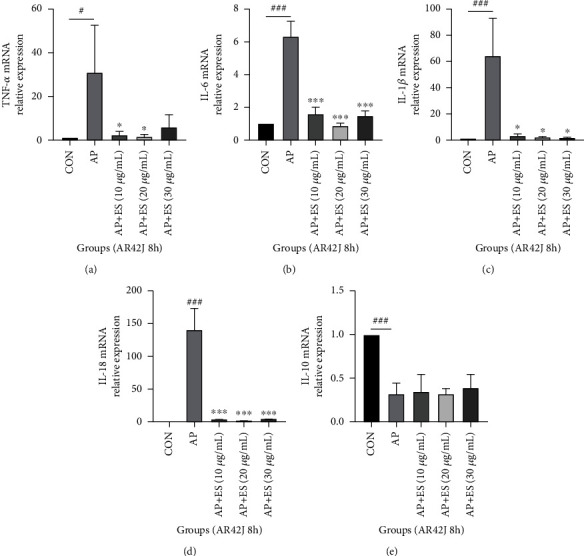
ES pretreatment inhibits inflammatory cytokines in vitro. AR42J cells were pretreated with different concentrations of ES for 1 h, then stimulated with STC for 4 h or 8 h. The data were presented as the mean ± SD of three replicate experiments. (a–d) Levels of TNF-*α*, IL-6, IL-1*β*, and IL-18 increased significantly in the AP group, and ES with different concentrations could downregulate them. (e) IL-10 decreased obviously in the AP group stimulated for 8 h, and ES pretreatment had no effect on it (^#^*P* < 0.05, ^##^*P* < 0.01, and ^###^*P* < 0.001 versus the CON group, ^∗^*P* < 0.05, ^∗∗^*P* < 0.01, and ^∗∗∗^*P* < 0.001 versus the AP group).

The process of acute pancreatitis is closely related to the release of inflammatory cytokines. We investigated the inflammatory cytokines by RT-qPCR to determine whether pretreated with ES could downregulate the expression of inflammatory cytokines in AP. TNF-*α*, IL-6, IL-1*β*, and IL-18 increased significantly in AP groups while ES pretreated groups attenuated production of TNF-*α*, IL-6, IL-1*β*, and IL-18 both in vivo and in vitro (Figures 3(a)–3(d), 4(a)–4(d), and 5(a)–5(d)). IL-10, an inhibitor of inflammatory response, decreased in the AP group in vivo but increased significantly in the ES (3 mg/kg) pretreatment group (Figure 3(e)). However, there were no obvious changes in cell experiments stimulated for 4 hours, but it decreased significantly in the AP group stimulated for 8 hours (Figures 4(e) and 5(e)).

These results demonstrate that ES pretreatment can effectively reduce the production of lipase, amylase, and inflammatory cytokines in STC-induced acute pancreatitis.

### 4.2. Escin Sodium Pretreatment Ameliorates Acinar Cell Injury in STC-Induced Acute Pancreatitis

**Figure 6:**
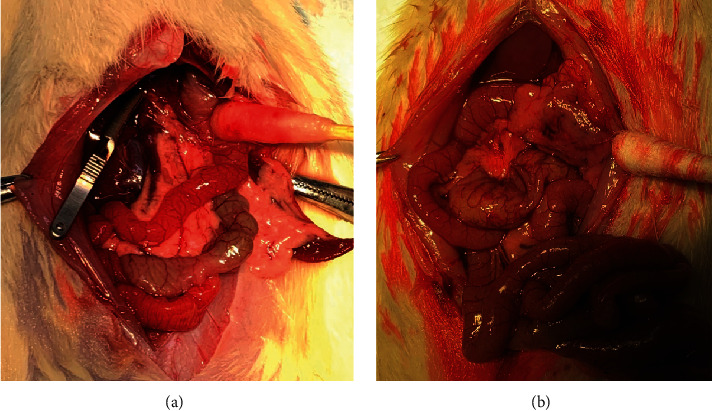
Morphological changes of the pancreas before and after modeling. (a) Shows a normal pancreas. (b) Shows the state after STC stimulation within 5 minutes; the pancreas indicated by the cotton swab presents ischemic necrosis.

**Figure 7:**
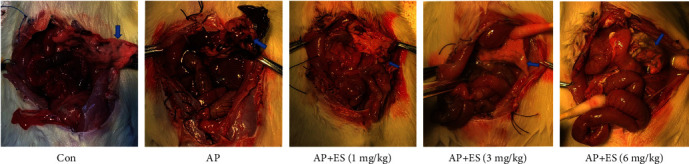
ES pretreatment alleviated pancreatic tissue injury in visual. The pancreas of the control group was almost normal. Ischemic necrosis and bloody ascites were shown in the AP group. The blue arrows indicate typical pancreatic tissue. The ES (3 mg/kg) pretreatment group was characterized by edema. The other ES pretreated groups (1 mg/kg and 6 mg/kg) had different degrees of tissue necrosis.

To control the quality of modeling, pancreatic tissues with 0.9% saline solution were used as contrast during the operation (Figure 6(a)). When edema and ischemia appeared in the tissues as shown in Figure 6(b), we thought the modeling was successful. 24 hours after the operation, we observed the pancreas. In the AP group, pancreatic tissues showed ischemic necrosis and bloody ascites in the abdominal cavity. However, the condition of the pancreas improved in ES pretreated groups compared with the AP group. As shown in Figure 7, the tissues suffered from less hemorrhage, necrosis, and bloody ascites in ES pretreatment groups. In the ES (3 mg/kg) pretreatment group, pancreatic tissues were mainly characterized by interstitial edema in contrast with ES (1 mg/kg, 6 mg/kg) pretreatment groups.

**Figure 8:**
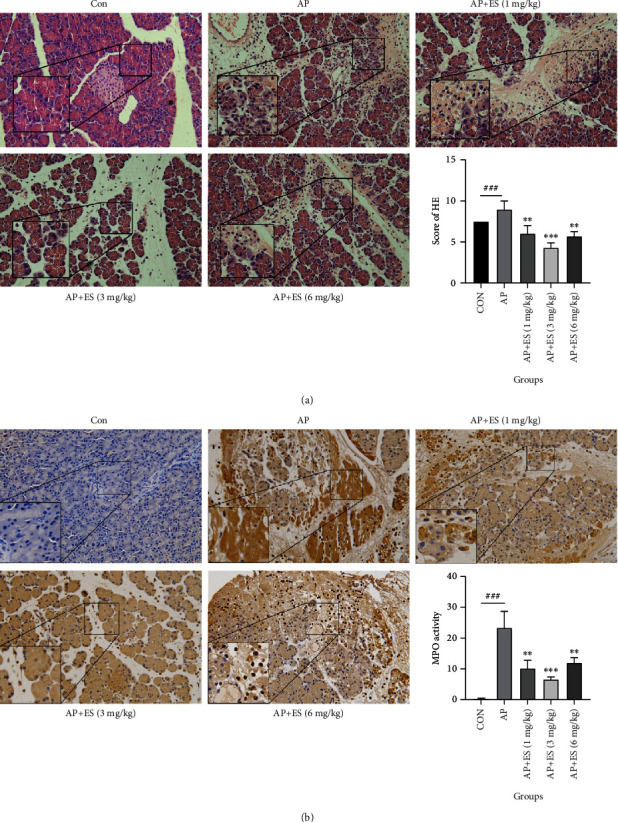
ES pretreatment effectively inhibits the acinar cell necrosis and reduces inflammatory cell infiltration and MPO level in vivo. (a) HE staining was used to evaluate the pancreatic pathological scores. Representative pictures from each group and quantitative analyses of histology score were shown. (b) The levels of MPO were further examined by immunohistochemistry. Representative images from each group and quantitative analyses of MPO were shown (original magnification, ×200) (^#^*P* < 0.05, ^##^*P* < 0.01, and ^###^*P* < 0.001 versus the CON group, ^∗^*P* < 0.05, ^∗∗^*P* < 0.01, and ^∗∗∗^*P* < 0.001 versus the AP group).

We assessed the HE staining score of tissues to determine whether ES pretreatment could ameliorate STC-induced AP in histomorphology. As shown in Figure 8(a), the histological features in the control group were normal, while in the AP group, histological features were characterized by edema, inflammatory cell infiltration, and necrosis, and the pathological score was significantly higher. However, compared with the AP group, characteristics of ES pretreated groups (1, 3, or 6 mg/kg) were mainly interstitial edema, with less necrosis and less infiltration of inflammatory cells, and the obviously lower pathological score. These results indicated that ES pretreatment could reduce the severity of acute pancreatitis induced by STC. Moreover, the ES (3 mg/kg) pretreatment group was more effective than the other two ES pretreated groups.

As a biomarker of activated neutrophils, MPO (myeloperoxidase) could be universally applied to evaluate the infiltration of neutrophils [27]. We furtherly evaluated MPO expression by immunohistochemical staining. In the control group, MPO was not detected while intense MPO immunostaining was shown in pancreatic acinar cells in the AP group, whereas pretreatment with different concentrations of ES could obviously downregulate the MPO levels (Figure 8(b)). In addition, the ES (3 mg/kg) pretreatment group had a better effect of reducing MPO level in contrast with the other two ES pretreated groups. These results were consistent with the HE staining. All these findings indicated that ES could ameliorate acinar cell injury in STC-induced acute pancreatitis.

### 4.3. Escin Sodium Promotes Acinar Cell Apoptosis but Not Pyroptosis in STC-Induced AP

**Figure 9:**
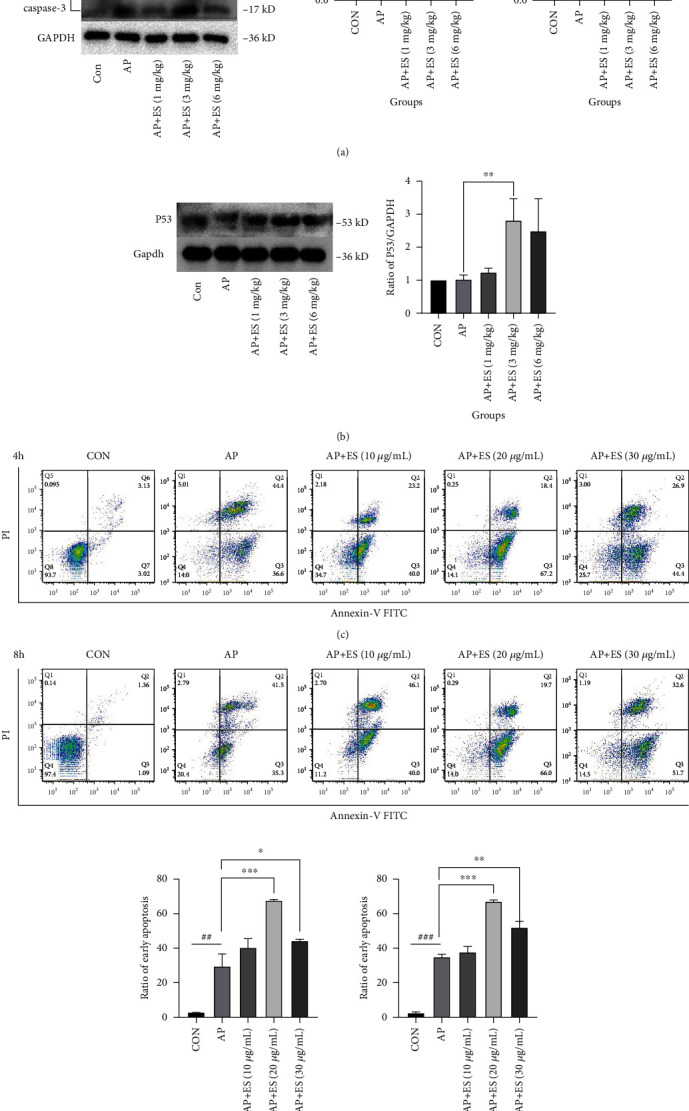
Effects of ES pretreatment on STC-induced apoptosis both in vivo and in vitro. (a, b) The expression of apoptotic-related proteins in different groups was determined by Western blotting: cleaved caspase-3, Bcl-2, Bax, and P53 in vivo. (c, d) Apoptosis of AR42J cells pretreated with different concentrations of ES for 1 h and then stimulated with STC for 4 h or 8 h, then examined using the Annexin-V-FITC/PI assay kit by flow cytometry. The ratio of early apoptosis (lower right quadrant) in different groups was quantified by FlowJo-V10 software (^#^*P* < 0.05, ^##^*P* < 0.01, and ^###^*P* < 0.001 versus the CON group, ^∗^*P* < 0.05, ^∗∗^*P* < 0.01, and ^∗∗∗^*P* < 0.001 versus the AP group).

The type of acinar cell death, like apoptosis, necrosis, and pyroptosis, will determine the prognosis of acute pancreatitis. Generally, it is believed that apoptosis is beneficial when acute pancreatitis onsets, as it cannot trigger the inflammatory cascade reaction. In contrast, necrosis or pyroptosis was understood as upregulators to damage response [8]. All the above results indicated that the ES pretreatment had protection effects in acute pancreatitis induced by STC, and we explored whether it was related to promoting apoptosis subsequently. We investigated the expression of cleaved caspase-3, Bcl-2, and Bax—biomarkers of apoptosis by Western blotting in vivo. Bcl-2 is an antiapoptotic marker. Bax, one of the members of the Bcl-2 family, is a proapoptotic effector protein that regulates the intrinsic pathway of apoptosis. The ratio of Bcl-2/Bax determines whether the cells are apoptotic or not. When the ratio is downregulated, apoptosis plays a crucial role and vice versa. As shown in Figure 9(a), the ratio of Bcl-2/Bax significantly decreased and cleaved caspase-3 evidently increased in the ES (3 mg/kg) pretreated group. P53 is a transcription factor that regulates the expression of essential apoptogenic factors, which cover both extrinsic and intrinsic apoptosis pathways [28, 29]. It is also directly interacted with Bcl-2 family members in a transcription-independent manner [30]. As shown in Figure 9(b), P53 was significantly upregulated in the ES (3 mg/kg) pretreatment group compared with the AP group.

To examine the promoting apoptotic effects of ES pretreatment on STC-induced AP, AR42J cells were pretreated with ES (10 *μ*g/mL, 20 *μ*g/mL, and 30 *μ*g/mL) for 1 h, and then STC was added for another 4 or 8 hours, respectively. We explored the apoptotic effect by flow cytometric analysis in vitro. As shown in Figures 9(c) and 9(d), the early apoptosis got increased in the ES (20 *μ*g/mL, 30 *μ*g/mL) pretreated group compared with the AP group. These results verified our hypothesis that ES pretreatment could ameliorate the prognosis of acinar cells in STC-induced AP by promoting apoptosis.

**Figure 10:**
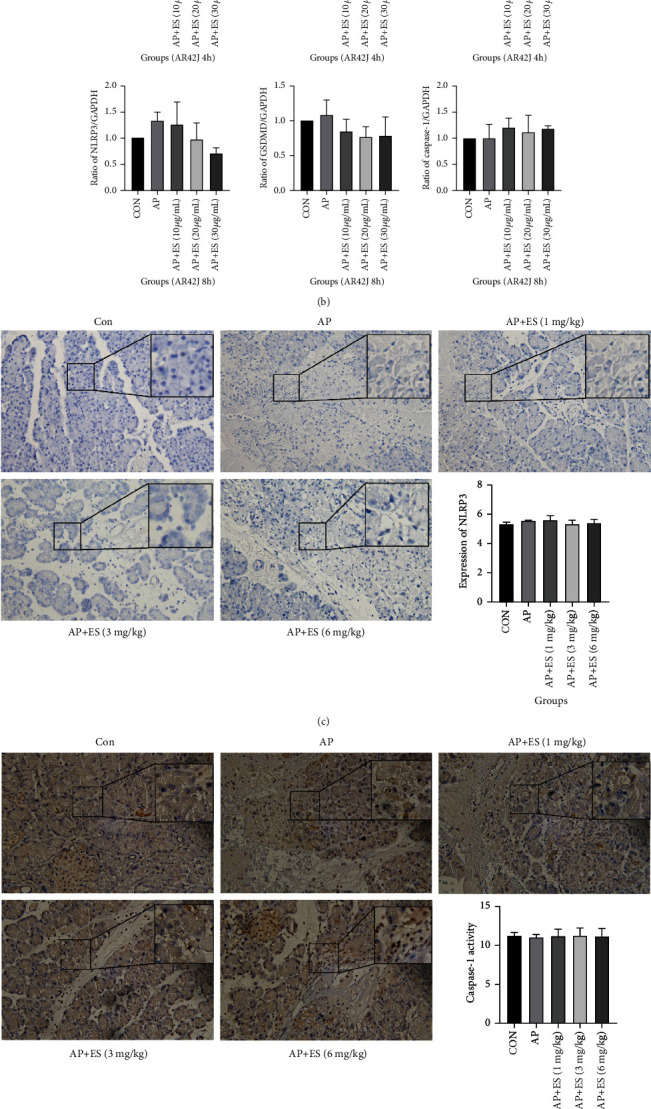
Expression of pyroptosis-related proteins in acinar cells in acute pancreatitis induced by STC in vivo and in vitro. (a, b) The expression of pyroptosis-related proteins: NLRP3, GSDMD, and caspase-1 were determined by Western blotting, and there was no significant difference in each group both in vivo and in vitro. (c–e) Levels of NLRP3, caspase-1, and GSDMD were examined by immunohistochemistry and showed no significant statistical difference, which further identified the Western blotting results. Representative pictures from each group and quantitative analyses of protein expression were shown (original magnification, ×200) (^#^*P* < 0.05, ^##^*P* < 0.01, and ^###^*P* < 0.001 versus the CON group, ^∗^*P* < 0.05, ^∗∗^*P* < 0.01, and ^∗∗∗^*P* < 0.001 versus the AP group).

Pyroptosis is one of programmed cell death that has received increased attention recently [31]. Studies have identified that caspase-1 is a mediator of pyroptosis and gasdermin D (GSDMD) is the executioner [32]. To determine whether pyroptosis is responsible for STC-induced acute pancreatitis, we detected NLRP3 (inflammasome sensor), caspase-1, and GSDMD expression by Western blotting in vivo and in vitro. We subsequently explored NLRP3, caspase-1, and GSDMD expressions by immunohistochemistry to further identify. As indicated in Figures 10(a) and 10(b), Western blotting analysis showed that there was no significant difference in pyroptosis proteins in each group, both in vivo and in vitro. What is more, the results of immunohistochemistry also verified these results (Figures 10(c)–10(e)) However, we observed the upregulation of pyroptosis proteins in individual cells by immunohistochemistry, even though it is not statistically significant.

### 4.4. Escin Sodium Plays a Protective Role by Upregulating Mitochondrial Membrane Potential and ROS Activity but Downregulating Intracellular Calcium Concentration

Mitochondria provide cellular energy and play a central role in regulating cell survival. Mitochondria are involved in cellular Ca^2+^ homeostasis and are a major source of ROS (reactive oxygen species) [33]. Mitochondrial membrane permeabilization (MMP, *ΔΨ*m) is a universal trigger of both necrosis and apoptosis. Loss of *ΔΨ*m leads to ATP (adenosine triphosphate—cell energy) depletion, ultimately leading to necrosis [34, 35]. The accumulation of MitoTracker Red CMXRos in the mitochondria depends on the *ΔΨ*m, so it is an indicator probe for the *ΔΨ*m. ROS usually play an important role in regulating apoptosis and critically regulate mitochondrial function and dysfunction [33, 36]. Ca^2+^ is a major regulator of the acinar cell function, but an abnormal (global and sustained) increase in cytosolic Ca^2+^ is a key pathologic signal associated with pancreatitis [37]. Abnormal Ca^2+^ signal promotes acinar cell necrosis through mitochondrial depolarization and subsequent ATP drop. To access apoptosis, mitochondrial function, ROS activity, and intracellular calcium concentration, Hoechst33342 staining, MitoTracker Red CMXRos staining, DCFH-DA, and Fluo-4-AM were used, respectively.

**Figure 11:**
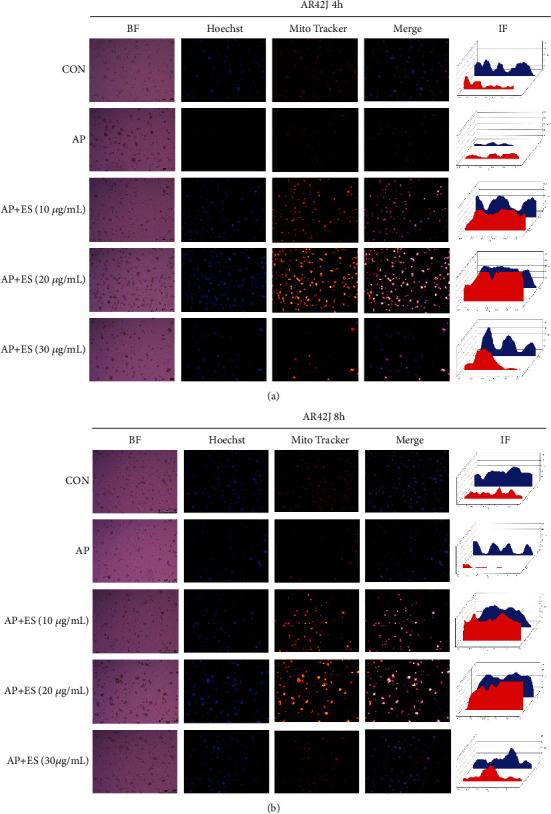
ES pretreatment induces apoptosis and increases mitochondrial membrane potential (MMP, *ΔΨ*m) in STC-induced acute pancreatitis. (a, b) AR42J cells were pretreated with different concentrations of ES for 1 h and then stimulated by STC for 4 h or 8 h, respectively. The fluorescence intensity was observed by fluorescence microscopy, and representative images are presented. Scale bar, 250 *μ*m. IF (intensity of fluorescence) was measured by ImageJ 6 times at different points randomly, then Origin2018 was used to show the IF, blue represents Hoechst32242, and red represents MitoTracker. BF: bright field; Hoechst: apoptosis staining; MitoTracker: *ΔΨ*m staining; merge: state after images overlay.

The fluorescence intensity of Hoechst33342 in ES pretreated groups was significantly higher than the AP group at 4 h and 8 h after stimulation (Figures 11(a) and 11(b)). It indicated that more apoptotic events occurred after ES pretreatment. *ΔΨ*m also increased significantly after ES pretreatment which was consistent with the results of Hoechst33342. However, compared with the control group, the fluorescence signals of Hoechst33342 and *ΔΨ*m in the AP group were downregulated.

**Figure 12:**
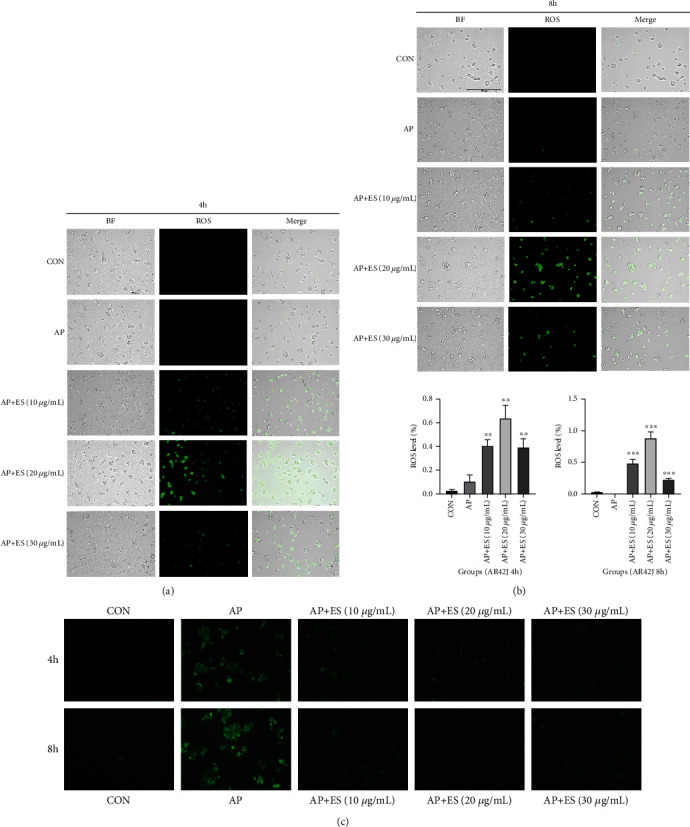
ES pretreatment triggered ROS generation in STC-induced acute pancreatitis. (a, b) AR42J cells were pretreated with different concentrations of ES for 1 h and then stimulated by STC for 4 h and 8 h, respectively. ROS generation was observed by fluorescence microscopy; representative images are presented. Scale bar, 250 *μ*m. BF: bright field; merge: state after images overlay. (c) Intracellular calcium concentration was observed by fluorescence microscopy, and representative images are presented. Scale bar, 100 *μ*m (^#^*P* < 0.05, ^##^*P* < 0.01, and ^###^*P* < 0.001 versus the CON group, ^∗^*P* < 0.05, ^∗∗^*P* < 0.01, and ^∗∗∗^*P* < 0.001 versus the AP group).

To clarify whether the protective effect of ES pretreatment is related to ROS and DCFH-DA, they were loaded to AR42J cells. As shown in Figures 12(a) and 12(b), in the control group, AR42J cells grew well without obvious green fluorescence. However, although there was no obvious fluorescence in the AP group, the cells showed lysis in BF (bright field), whereas ES pretreatment groups showed strong fluorescence signal no matter they were stimulated for 4 hours or 8 hours, and the difference was statistically significant. Interestingly, the level of ROS is positively correlated with change of *ΔΨ*m.

And then, we furtherly explored intracellular calcium concentration by Fluo-4-AM working solution. As shown in Figures 12(c), strong fluorescence signals in the AP group were detected compared with the control group. However, ES pretreatment could downregulate the fluorescence signal intensity. The results suggested that intracellular calcium overloaded in the AP group, and ES pretreatment can downregulate intracellular calcium concentration, which is opposite to ROS and *ΔΨ*m.

### 4.5. Escin Sodium Promotes Acinar Cell Apoptosis via Downregulating ERK/STAT3 Phosphorylation

**Figure 13:**
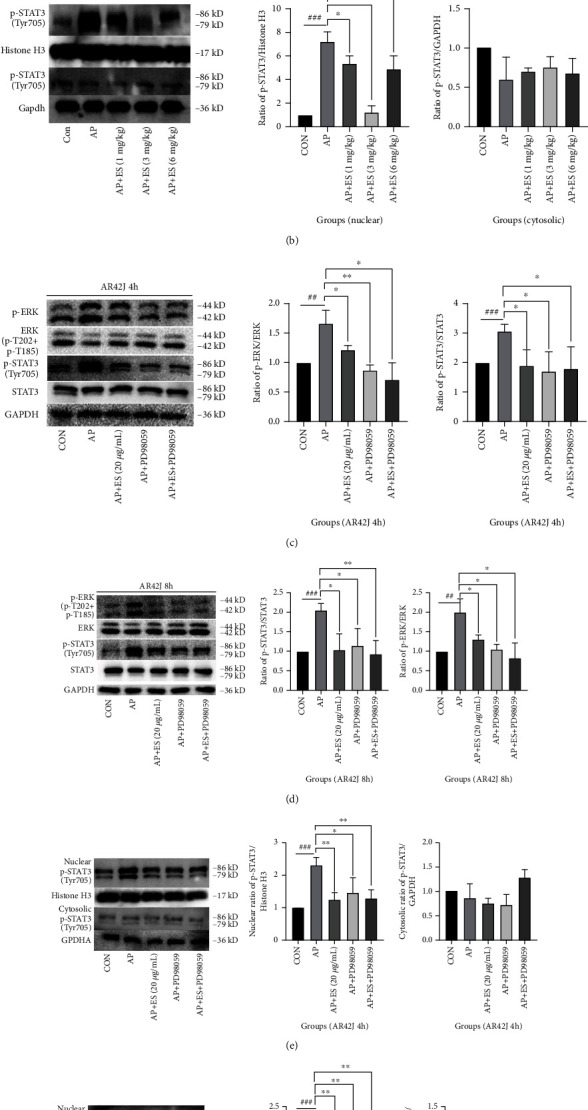
Roles of the ERK/STAT3 signaling pathway in STC-induced acute pancreatitis. (a) The expression levels of p-ERK, ERK, p-STAT3, and STAT3 were analyzed by Western blotting in vivo. (b) The expression of p-STAT3 in nuclears of the AP group was increased, while it was decreased after pretreatment with ES. However, there was no significant change in p-STAT3 in the cytoplasm. (c, d) Cells were pretreated with ES (20 *μ*g/mL), ERK inhibitor PD98059 (10 *μ*M), and ES (20 *μ*g/mL)+ PD98059 (10 *μ*M) for 1 h, then stimulated by STC for 4 h or 8 h, respectively. The expression levels of p-ERK, ERK, p-STAT3, and STAT3 were analyzed by western blotting in vitro. ES could significantly downregulate the ERK/STAT3 signaling pathway. (e, f) The expression of p-STAT3 in nuclears of the AP group was increased, while it was decreased after pretreatment with ES, PD98059, and ES+PD98059. However, there was no significant change in p-STAT3 in the cytoplasm in each group (^#^*P* < 0.05, ^##^*P* < 0.01, and ^###^*P* < 0.001 versus the CON group, ^∗^*P* < 0.05, ^∗∗^*P* < 0.01, and ^∗∗∗^*P* < 0.001 versus the AP group).

The above results suggested that ES pretreatment could maintain a higher level of *ΔΨ*m and ROS, downregulate intracellular calcium concentration, and promote early apoptosis in STC-induced acute pancreatitis. To elucidate the mechanism of ES promoting apoptosis in AP induced by STC, we hypothesized that it is related to ERK/STAT3/Bcl-2 axis. To verify our hypothesis, we first analyzed p-ERK/ERK and p-STAT3/STAT3 in vivo by Western blotting. As shown in Figure 13(a), ES pretreated groups (1 mg/kg, 3 mg/kg) dramatically downregulated the expressions of p-ERK and p-STAT3. We then extracted the cytoplasmic and nuclear proteins from the tissue and found that the nuclear expression of p-STAT3 was increased in the AP group, while decreased obviously in ES pretreatment groups. Interestingly, however, there was no significant change in the expression of p-STAT3 in the cytoplasm (Figure 13(b)).

We further tested with pathway inhibitor—PD98059 in vitro. According to the above results, compared with the ES (10 *μ*g/mL, 30 *μ*g/mL) pretreated groups, ES (20 *μ*g/mL) pretreatment has more advantages in promoting apoptosis. We then divided AR42J cells into 5 groups: control group, AP group, AP+ES (20 *μ*g/mL) group, AP+PD98059 (10 *μ*M) group, and AP+PD98059(10 *μ*M)+ES (20 *μ*g/mL) group (combined group). We detected relevant cellular signaling pathway proteins by Western blotting. In the AP group, p-ERK and p-STAT3 were activated in contrast with the control group (Figures 13(c) and 13(d)). However, ERK phosphorylation was significantly downregulated after ES pretreatment and the same trend also appeared in STAT3 phosphorylation. We then extracted the cytoplasmic and nuclear proteins from the cells and found that p-STAT3 in the nucleus was increased considerably in the AP group and decreased obviously in the ES pretreated group. But there was still no apparent change in p-STAT3 in the cytoplasm. This was synchronized with the results of ERK inhibitors. Moreover, the results were consistent no matter whether the stimulation lasted for 4 hours or 8 hours (Figures 13(e) and 13(f)).

**Figure 14:**
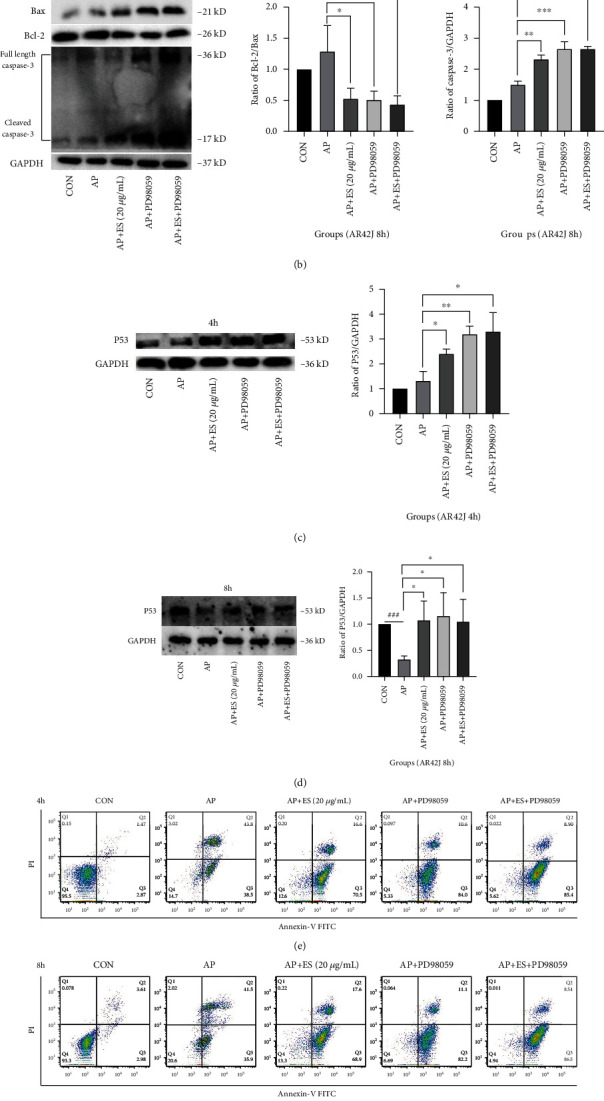
ES promotes apoptosis in STC-induced acute pancreatitis via downregulating the ERK/STAT3 pathway. (a-d) AR42J cells were pretreated with ES (20 *μ*g/mL), ERK inhibitor PD98059 (10 *μ*M), and ES (20 *μ*g/mL)+ PD98059 (10 *μ*M) for 1 h, then stimulated by STC for 4 h or 8 h, respectively. The expression levels of cleaved caspase-3, Bcl-2, Bax, and P53 were analyzed by Western blotting. (e, f) Apoptosis of AR42J cells in each group was examined using the Annexin-V-FITC/PI assay kit by flow cytometry. The ratio of early apoptosis (lower right quadrant) in different groups was quantified by FlowJo-V10 software (^#^*P* < 0.05, ^##^*P* < 0.01, and ^###^*P* < 0.001 versus the CON group, ^∗^*P* < 0.05, ^∗∗^*P* < 0.01, and ^∗∗∗^*P* < 0.001 versus the AP group).

To further clarify the mechanisms of the ERK/STAT3 pathway and apoptosis, we then measured the expression of apoptotic-related proteins—Bcl-2, Bax, cleaved caspase-3, and P53. The early apoptosis of AR42J cells in each group was detected again by flow cytometry. As shown in Figures 14(a)–14(d), cleaved caspase-3 and P53 were upregulated apparently in ES (20 *μ*g/mL), PD98059, and combined groups and were statistical significant in contrast with the AP group. Moreover, the ratio of Bcl-2/Bax showed the opposite trend. Flow cytometry analysis showed that the ES pretreatment group could promote early apoptosis as well as the PD98059 group. Moreover, the combined group showed a better trend of promoting apoptosis (Figures 14(e) and 14(f)). Taken all the results into consideration, we deduced that ES promotes acinar cell apoptosis via downregulating ERK and STAT3 phosphorylation.

## 5. Discussion

Escin Sodium (ES) has been commonly used in clinical, as its anti-inflammatory and antiedematous properties make it a choice of therapy for chronic venous insufficiency. Nowadays, ES has also been investigated against diverse cancers [38]. However, only little is known about the effect of ES on acute pancreatitis, and the corresponding mechanism remains obscure.

In this study, we confirmed that ES exerts a protective effect in STC-induced AP by promoting apoptosis through the inactivation of the ERK/STAT3 signaling pathway. AP induced by STC is a reliable and reproducible model with a short duration. When acute pancreatitis onsets, the increased permeability of acinar cells produces serum amylase and lipase that release to circulation at high levels [2]. The practice guidelines released by the ACG (American College of Gastroenterology) suggested that both serum amylase and lipase could be applied for the diagnosis of AP [39]. Our study demonstrated that STC significantly increased serum amylase and lipase, whereas ES pretreatment showed conversed outcomes dramatically in vivo. In vitro study, STC only increased amylase in cell supernatant rather than lipase. We do not know why there is no obvious change in lipase, which needs further study. However, amylase decreased significantly after ES pretreatment in vitro.

As mentioned above, acute pancreatitis not only causes local inflammations but also causes systemic inflammatory responses, which can induce serious complications such as MODS or SIRS. Various pathological factors that lead to acinar cell injury stimulate inflammatory responses (infiltration of neutrophils, release of cytokines TNF-*α* and IL-6, etc.) [40]. Suppression of proinflammatory cytokines could ameliorate the severity of pancreatitis. Our study has shown that TNF-*α*, IL-6, IL-1*β*, and IL-18 decreased significantly after ES pretreatment both in vivo and in vitro. IL-10 has been considered as an anti-inflammatory cytokine which contributes to the maintenance of immune homeostasis [41]. We explored whether the function of ES is related to the increase of IL-10. The results indicated that IL-10 in the AP group was significantly lower than in the control group, whereas ES (3 mg/kg) pretreatment could increase it obviously. In vitro study, there was no significant change in IL-10 in each group stimulated for 4 hours, but with the extension of stimulation (8 hours), the level of IL-10 in the AP group decreased significantly. It was not completely consistent in vivo and in vitro experiments. Based on this phenomenon, we hold the opinion that the environment of the body is complex, and cells in vitro cannot be fully simulated.

To further assess the effects of ES pretreatment on pathological features in STC-induced acute pancreatitis, HE staining and MPO activity were used for evaluation. The results suggested that ES could alleviate acinar cell damage, reduce inflammatory cell infiltration, and downregulate the expression of MPO.

Apoptosis, a pattern of programmed cell death, is considered to be a benefit to the prognosis of acute pancreatitis. In our study, we found that ES pretreatment activated apoptotic relevant proteins in rats, and then we detected apoptosis by flow cytometry in AR42J cells. As indicated by our results, ES pretreatment promoted early apoptosis. Pyroptosis, another pattern of programmed cell death, is considered to be involved in the process of acute pancreatitis. In the present study, we first identified pyroptosis-related proteins in the pancreas and in AR42J cells. NLRP3/caspase-1/GSDMD were detected by Western blotting and immunohistochemistry. According to the above results, both the Western blotting and the immunohistochemical results suggested that there was no significant difference in pyroptosis-related proteins. However, it was worth noting that high expression of pyroptosis proteins was found in individual cell by immunohistochemistry. Hence, we deduce that pyroptosis does exist when acute pancreatitis onsets, but it is not the main way of death in acinar cells induced by STC.

Mitochondria, positioned at the heart of cellular metabolism [34], and MMP (*ΔΨ*m) play a decisive role in determining the way of cell death. Loss of *ΔΨ*m leads to ATP depletion, unable to maintain ionic gradients across the plasma membrane, causing necrosis finally. Mitochondria also commit the apoptotic pathway by releasing the resident protein cytochrome c and lead to the downstream apoptotic events [33, 35, 42]. All the above results have identified the effect of ES pretreatment on promoting apoptosis; we then further explored the mitochondrial function and ROS activity to fully understand its apoptotic-related events. In the present study, we found that STC caused the loss of *ΔΨ*m, whereas ES pretreatment enhanced it. The fluorescence intensity of Hoechst32242 was attenuated in the AP group but increased in ES pretreatment groups, which were consistent with the changes of *ΔΨ*m. Therefore, we speculate that ES pretreatment promoted apoptosis by changing the mitochondrial membrane potential.

ROS mainly originate from mitochondria, but little was known about its properties and their role in cell death in acute pancreatitis. We next detected ROS activity in AR42J cells. As shown in the above results, ES pretreatment can increase ROS activity, which is consistent with the changes of *ΔΨ*m and Hoechst32242. A study [43] by Booth et al. has proved that ROS exert its protective effect in acinar cells, which is different from many other cell types. As for why ROS are harmful to other cells (e.g., liver cells) and beneficial to pancreatic acinar cells, we are still uninformed.

In pancreatic mitochondria, supramaximal CCK, cerulein, or TLC-S cause Ca^2+^-dependent loss of *ΔΨ*m. *ΔΨ*m loss caused by abnormal Ca^2+^ signal not only promotes necrosis but also inhibits apoptosis by limiting cytochrome c release [44]. Our results showed that intracellular calcium overloaded in the AP group, which was consistent with the literatures [45]. However, the group pretreated with different concentrations of ES could reduce the intracellular calcium concentration in different degrees. Generally, the decrease of *ΔΨ*m and ROS and the increase of calcium concentration exist at the same time when acute pancreatitis onsets, which can be reversed by ES pretreatment. However, the relationships between calcium concentration, ROS, and *ΔΨ*m in acinar cells are interesting and blurred and need further exploration.

Apoptosis preserves the plasma membrane integrity and is thus considered to be immunologically silent. This is essentially different from cell necrosis. Necrotic cell suffers from serious cell membrane rupture, causing inflammatory cascade reactions [46]. The expression of Bcl-2 and Bax is essential for *ΔΨ*m changes, and P53 can move to mitochondria which directly promotes MOMP (mitochondrial outer membrane permeabilization), inducing apoptosis. Hu et al. showed that transcriptional activity of p53 was impaired, which explained the apoptosis silencing during AP [29]. Our results show that ES pretreatment can induce apoptosis in AP by activating caspase-3 and P53 and downregulating the Bcl-2/Bax ratio. Taken all the results into consideration, we suppose that ES may regulate *ΔΨ*m by changing Bcl-2/Bax and P53, thus promoting apoptosis. But this mechanism is complicated, appealing for deeper research.

To elucidate the upstream of ES on apoptotic pathways, we furtherly explored a potential one—ERK/STAT3—which might be involved in this process. In the classic MAPK cascade activation pathway, ERK plays an important role and its phosphorylation can affect many cellular functions, including apoptosis [21, 47]. STAT3 (signal transducer and activator of transcription 3) has also been linked with survival, proliferation, etc. [20]. P-ERK (phosphorylation of ERK) is closely related to p-STAT3 (phosphorylation of STAT3) [21], while Bcl-2, one of the STAT3 target genes, is involved in mitochondrial-related cell death. We first found that ES could reduce the level of p-ERK and p-STAT3 consistently in vivo in STC-induced acute pancreatitis. Then, we blocked the activation of ERK with PD98059 in AR42J cells to further confirm the relationship between ERK/STAT3 and apoptosis. As shown in the above results, the ES (20 *μ*g/mL) pretreatment group could significantly downregulate p-ERK and p-STAT3 simultaneously with PD98059, but when used in combination, it did not further reduce. Activation of caspase-3 and Bax are positively correlated with apoptosis [48], while the ratio of Bcl-2/Bax is negatively correlated with it. We next found that both ES (20 *μ*g/mL) and PD98059 could upregulate cleaved caspase-3 and P53 and downregulate the Bcl-2/Bax ratio apparently. What is more, when applied in combination, they furtherly promoted apoptosis. The results of flow cytometry are also consistent with those of Western blotting.

## 6. Conclusion

**Figure 15:**
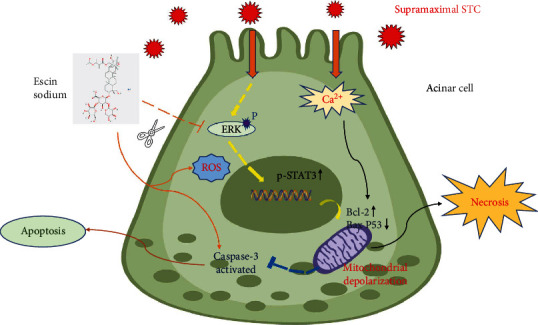
A brief diagram of the effects of ES on pancreatic acinar cells.

In summary, Escin Sodium pretreatment improves the prognosis of acute pancreatitis induced by STC via inducing apoptosis mediated by the ERK/STAT3 signaling pathway (Figure 15). The results of this study provide new insight into the potential efficacy of Escin Sodium in the treatment of acute pancreatitis.

## Figures and Tables

**Table 1:** Histological scoring for acute pancreatitis.

Condition	Score	Description
Edema	0	Absent
	1	Diffuse expansion of interlobular septa
	2	1+ diffuse expansion of interlobular septa
	3	2+ diffuse expansion of interlobular septa
	4	3+ diffuse expansion of intercellular septa

Inflammation (%)	0	Absent
	1	Around ductal margin
	2	In parenchyma (<50 of lobules)
	3	In parenchyma (51-75 of lobules)
	4	In parenchyma (>75 of lobules)

Vacuolization (%)	0	Absent
	1	Periductal (<5)
	2	Focal (5-20)
	3	Diffuse (21-50)
	4	Severe (>50)

Cell necrosis	0	Absent
	1	<10% necrosis
	2	<40% necrosis
	3	>40%necrosis
	4	>60%necrosis

## Data Availability

The data that support the findings of this study are available from the corresponding author upon reasonable request.

## Abbreviations

AP: Acute pancreatitis
ES: Escin Sodium
MMP, *ΔΨ*m: Mitochondrial membrane potential
ROS: Reactive oxygen species
SAP: Severe acute pancreatitis
MODS: Multiple organ dysfunction syndrome
SIRS: Systemic inflammatory response syndrome
STAT3: Signal transducer and activator of transcription
ERK: Extracellular regulated protein kinases
FBS: Fetal bovine serum
PBS: Phosphate buffered saline
Bcl-2: B-cell lymphoma 2
Bax: Bcl2-associated X protein
Caspase-3: Cysteine aspartic acid protease 3
MOMP: Mitochondrial outer membrane permeabilization.

## Supplementary Materials

Supplementary Materials Concise supplementary material description: (a) modeling method. (b) The state of rats in the model group after modeling. (c) The state of rats in the ES pretreated group after modeling. (d) Ascites status of model group. (e–g) Ascites status of ES pretreated groups (1 mg/kg, 3 mg/kg, and 6 mg/kg).
