# Supplementary figures and images for: Escin Sodium Improves the Prognosis of Acute Pancreatitis via Promoting Cell Apoptosis by Suppression of the ERK/STAT3 Signaling Pathway
Source: Oxid Med Cell Longev. 2021 Aug 12;2021:9921839. doi: 10.1155/2021/9921839 (PMC8378969; doi:10.1155/2021/9921839)

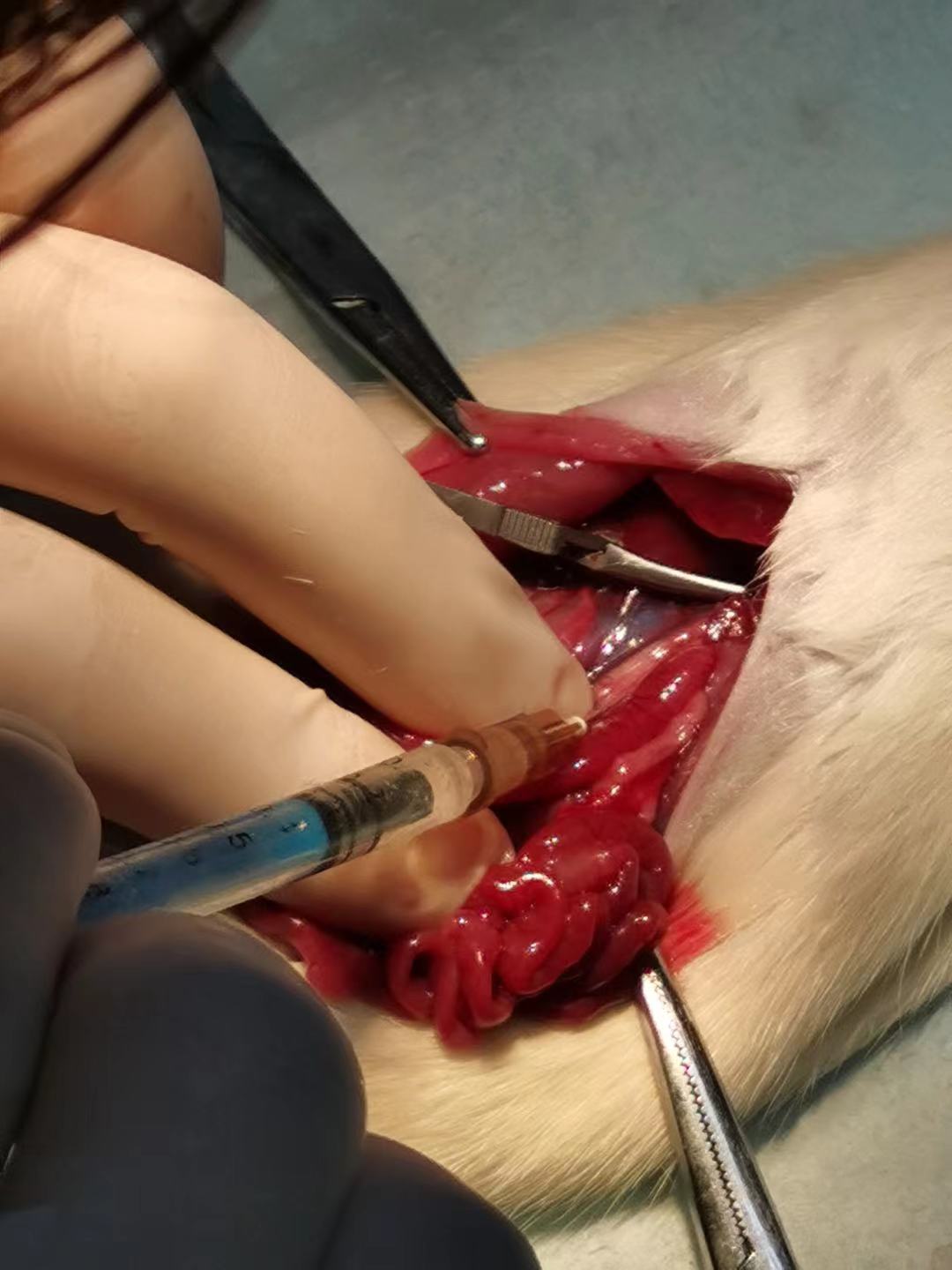

Supplement: Supplementary Materials — Concise supplementary material description: (a) modeling method. (b) The state of rats in the model group after modeling. (c) The state of rats in the ES pretreated group after modeling. (d) Ascites status of model group. (e–g) Ascites status of ES pretreated groups (1 mg/kg, 3 mg/kg, and 6 mg/kg). [file 9921839.f1.zip › a.jpg]

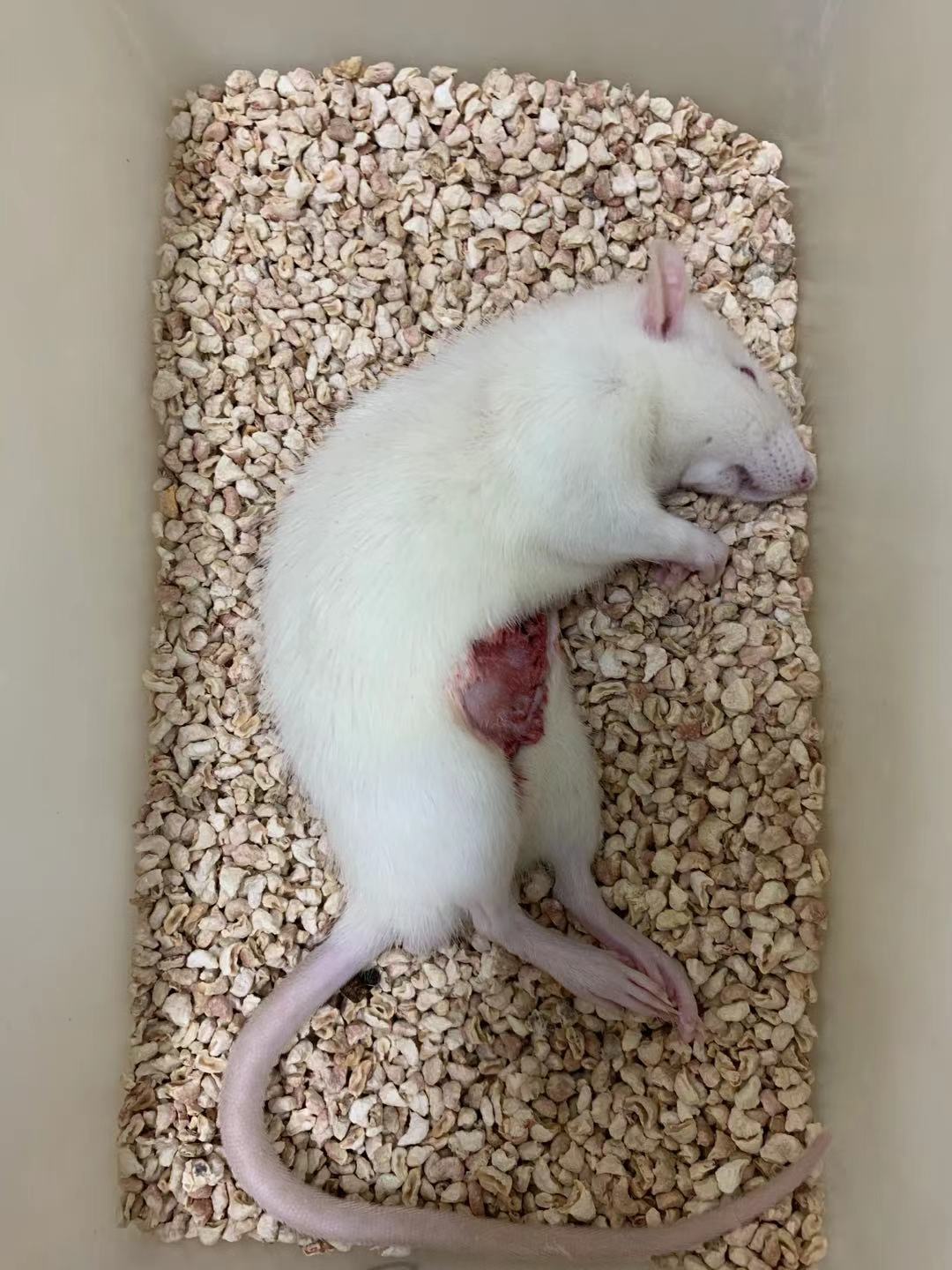

Supplement: Supplementary Materials — Concise supplementary material description: (a) modeling method. (b) The state of rats in the model group after modeling. (c) The state of rats in the ES pretreated group after modeling. (d) Ascites status of model group. (e–g) Ascites status of ES pretreated groups (1 mg/kg, 3 mg/kg, and 6 mg/kg). [file 9921839.f1.zip › b.jpg]

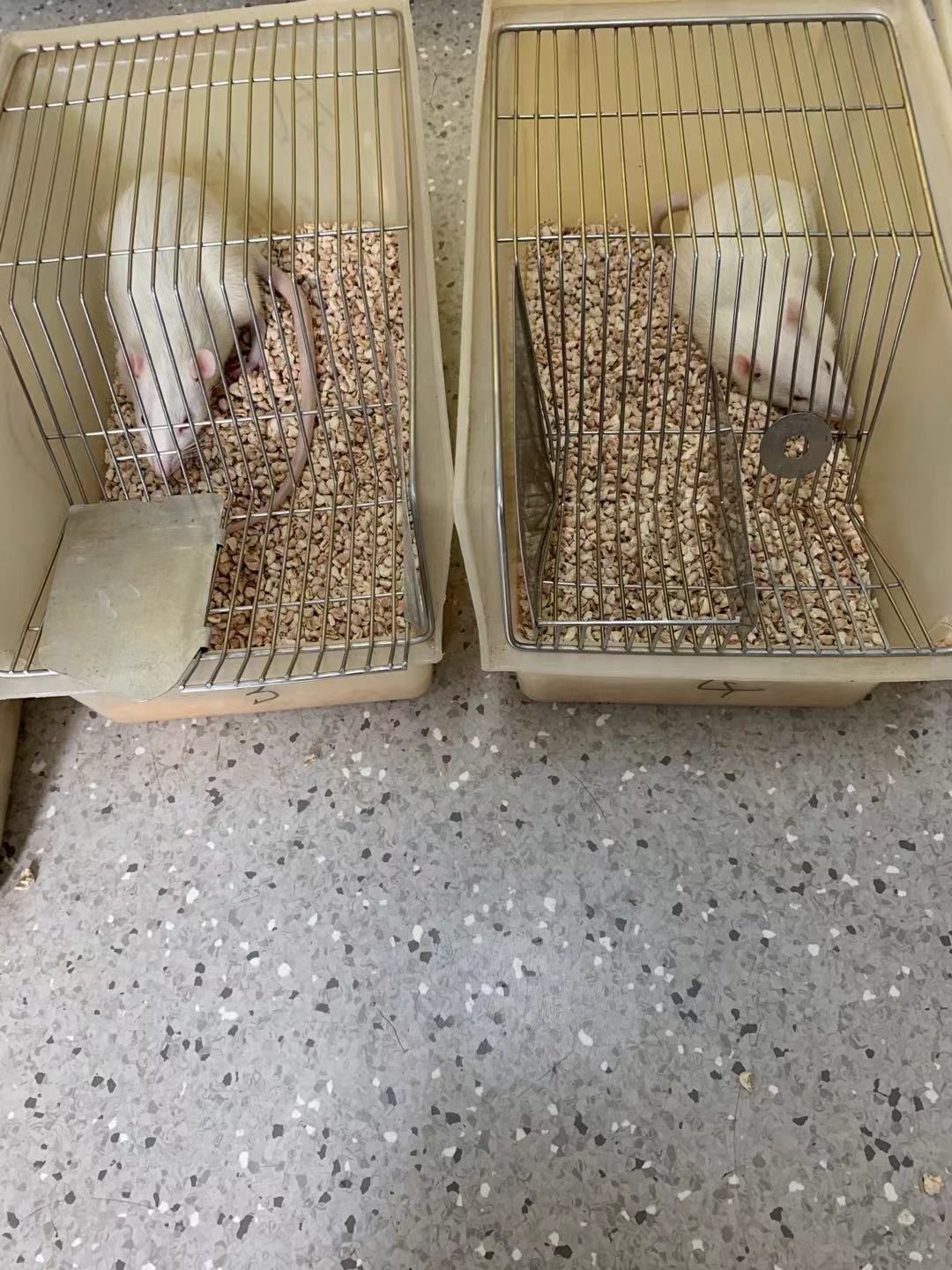

Supplement: Supplementary Materials — Concise supplementary material description: (a) modeling method. (b) The state of rats in the model group after modeling. (c) The state of rats in the ES pretreated group after modeling. (d) Ascites status of model group. (e–g) Ascites status of ES pretreated groups (1 mg/kg, 3 mg/kg, and 6 mg/kg). [file 9921839.f1.zip › c.jpg]

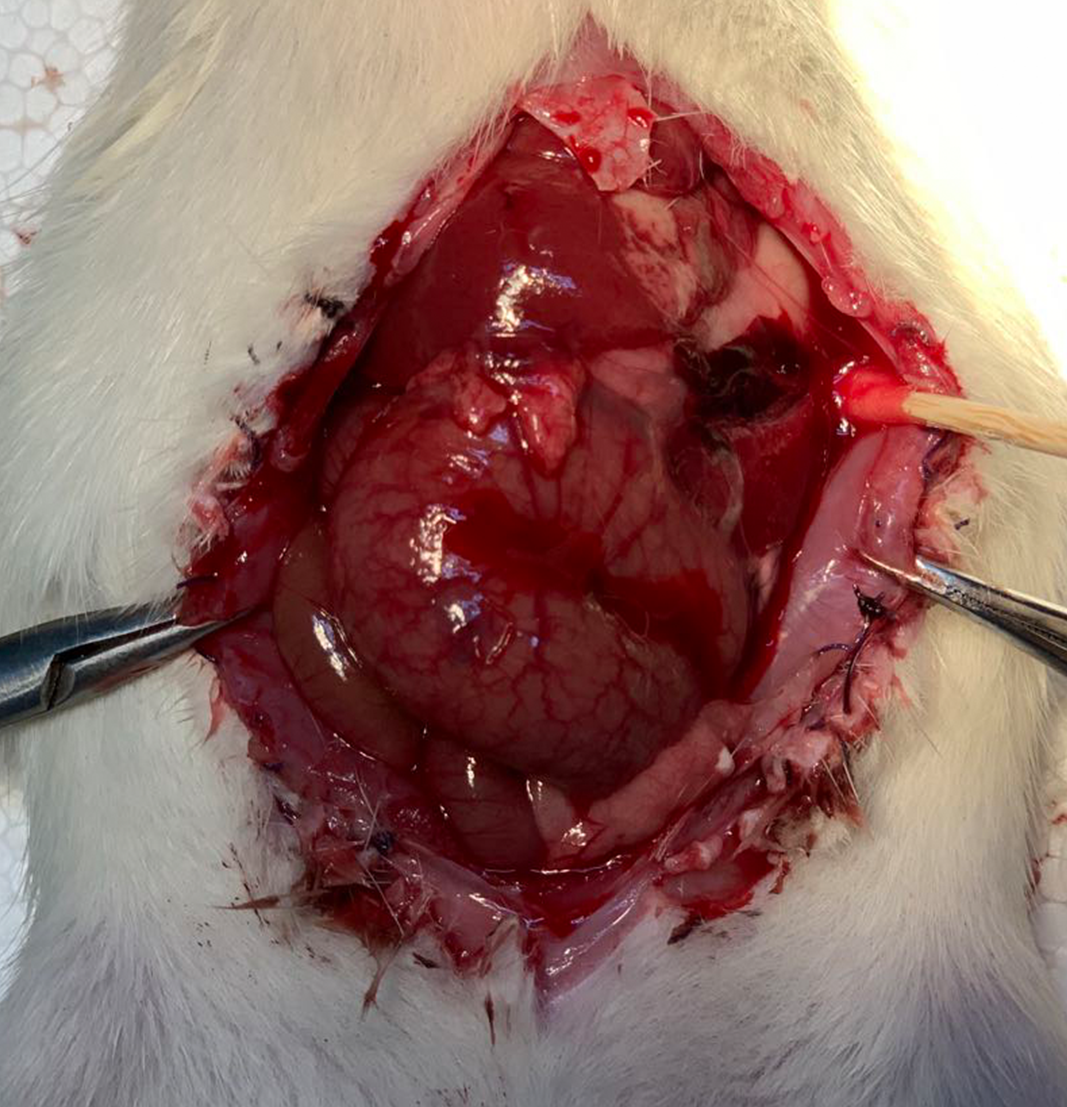

Supplement: Supplementary Materials — Concise supplementary material description: (a) modeling method. (b) The state of rats in the model group after modeling. (c) The state of rats in the ES pretreated group after modeling. (d) Ascites status of model group. (e–g) Ascites status of ES pretreated groups (1 mg/kg, 3 mg/kg, and 6 mg/kg). [file 9921839.f1.zip › d.png]

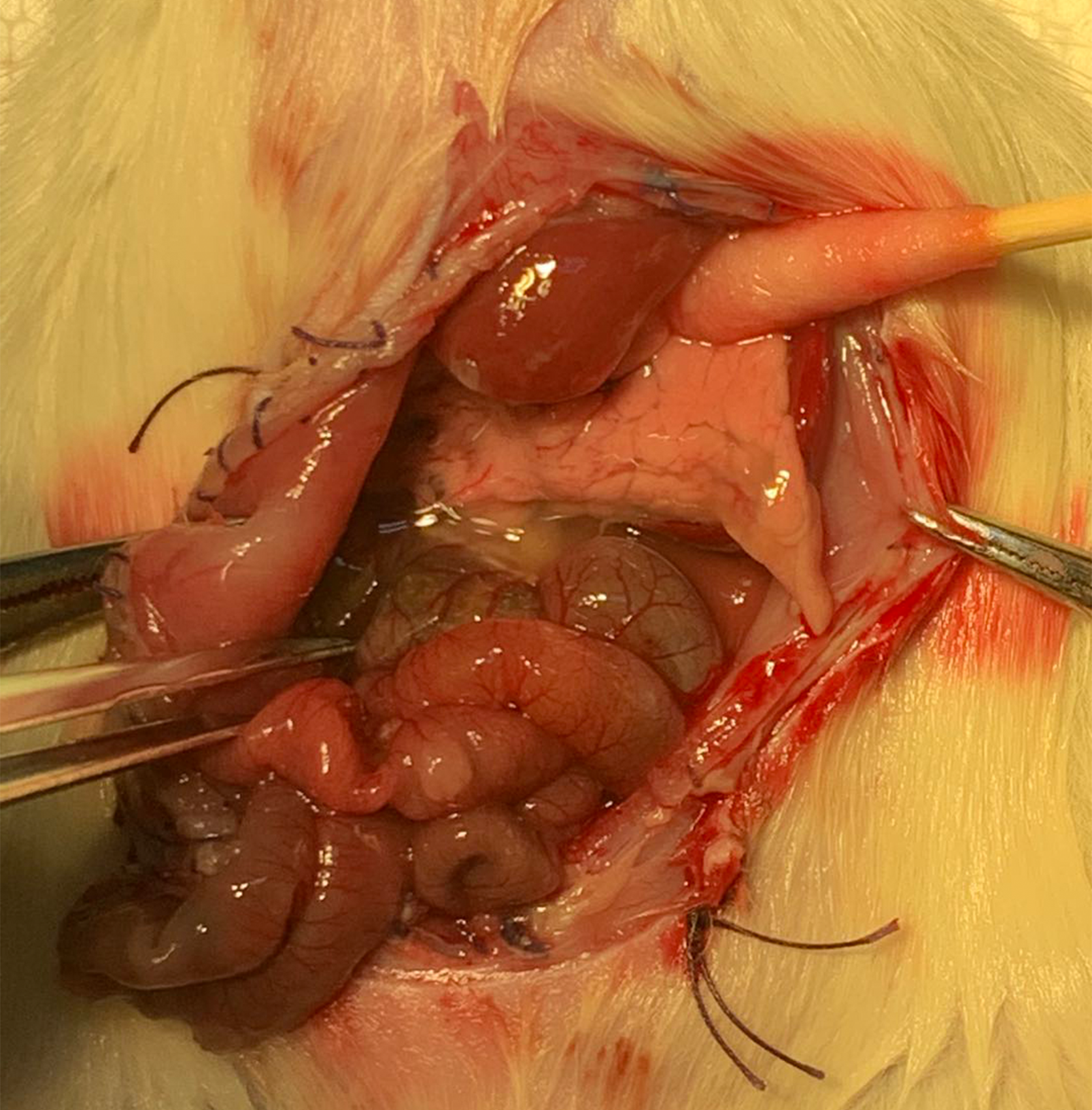

Supplement: Supplementary Materials — Concise supplementary material description: (a) modeling method. (b) The state of rats in the model group after modeling. (c) The state of rats in the ES pretreated group after modeling. (d) Ascites status of model group. (e–g) Ascites status of ES pretreated groups (1 mg/kg, 3 mg/kg, and 6 mg/kg). [file 9921839.f1.zip › e.png]

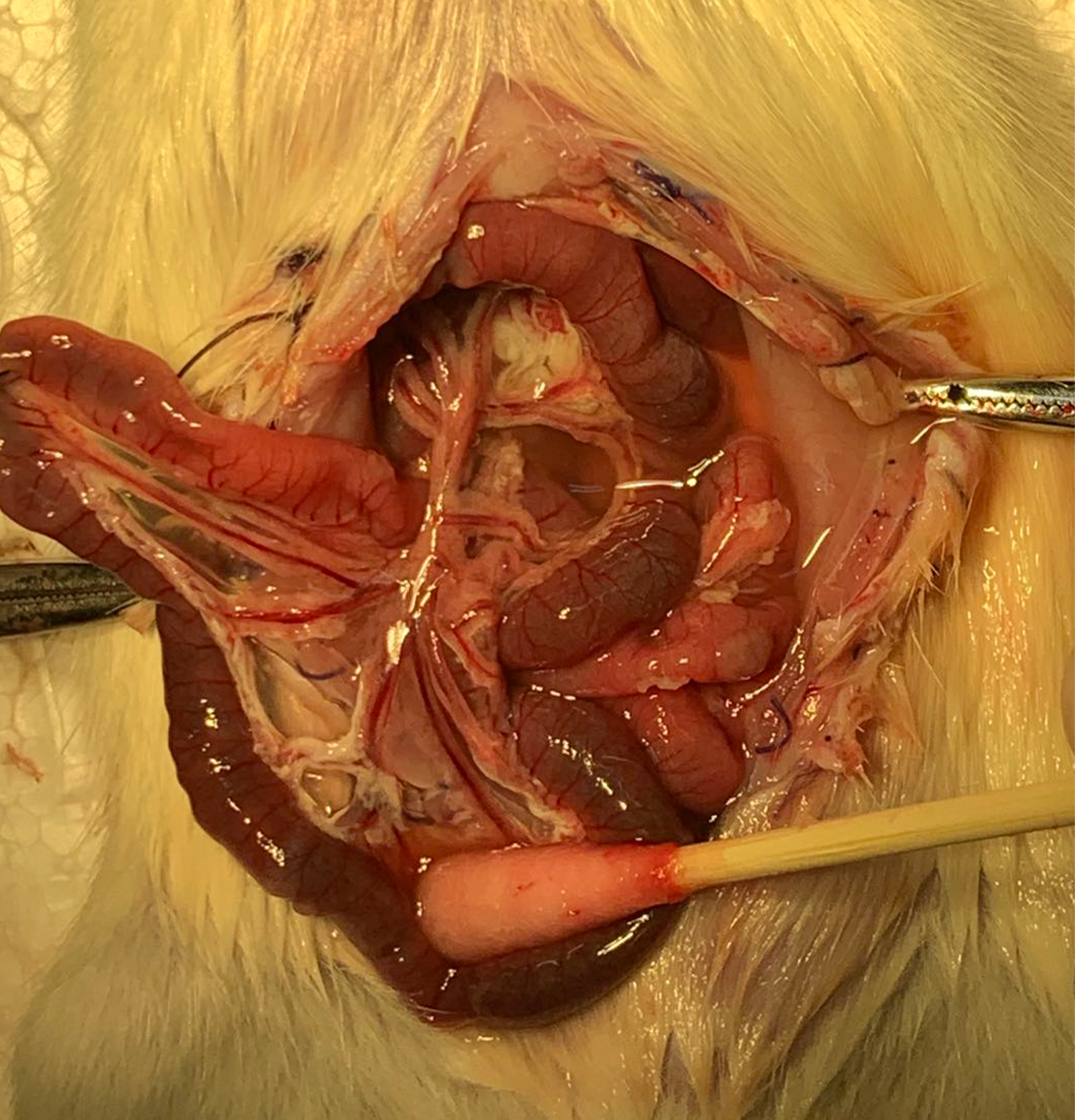

Supplement: Supplementary Materials — Concise supplementary material description: (a) modeling method. (b) The state of rats in the model group after modeling. (c) The state of rats in the ES pretreated group after modeling. (d) Ascites status of model group. (e–g) Ascites status of ES pretreated groups (1 mg/kg, 3 mg/kg, and 6 mg/kg). [file 9921839.f1.zip › f.png]

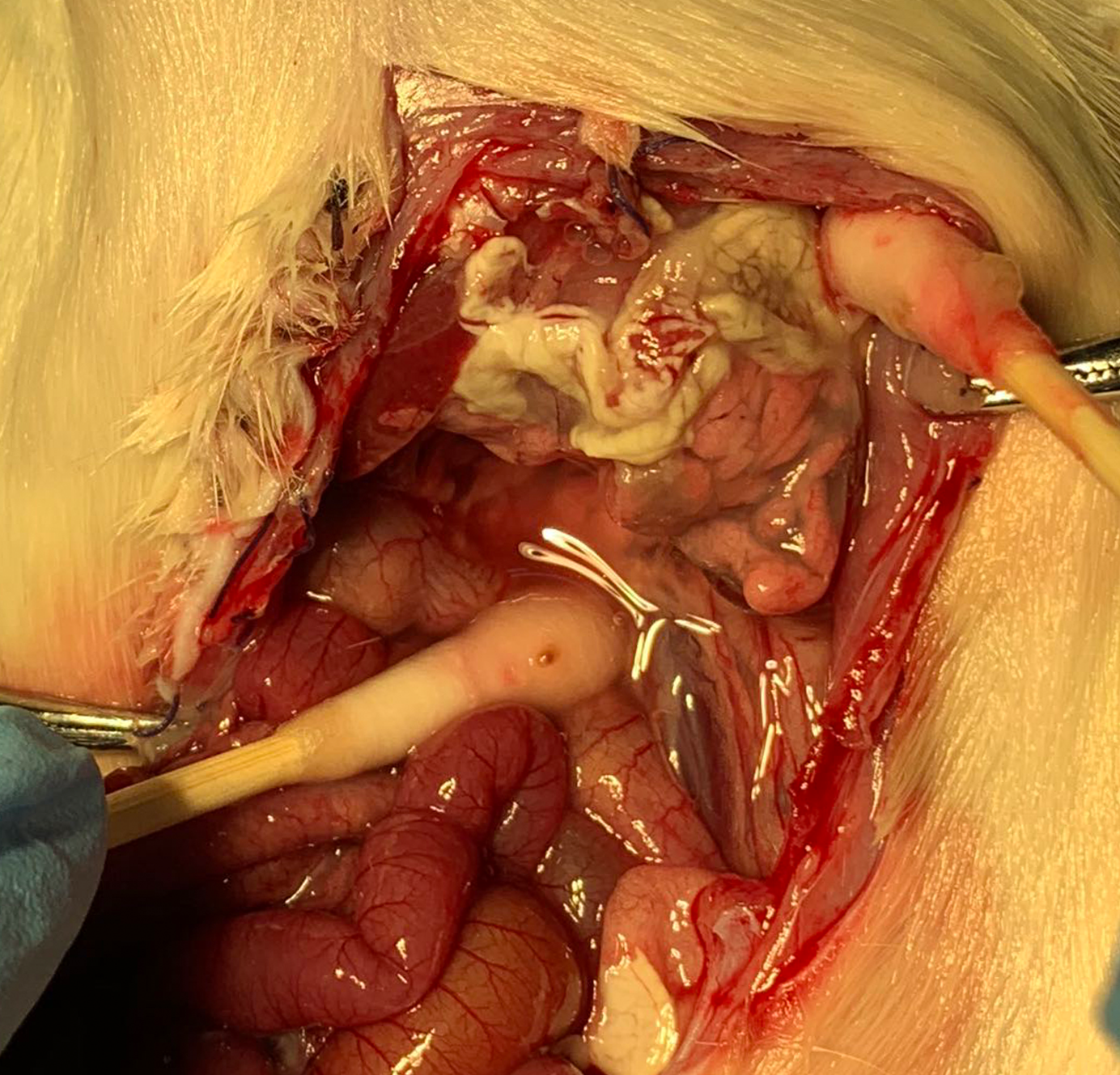

Supplement: Supplementary Materials — Concise supplementary material description: (a) modeling method. (b) The state of rats in the model group after modeling. (c) The state of rats in the ES pretreated group after modeling. (d) Ascites status of model group. (e–g) Ascites status of ES pretreated groups (1 mg/kg, 3 mg/kg, and 6 mg/kg). [file 9921839.f1.zip › g.png]
